# NINJA-associated ERF19 negatively regulates Arabidopsis pattern-triggered immunity

**DOI:** 10.1093/jxb/ery414

**Published:** 2018-11-21

**Authors:** Pin-Yao Huang, Jingsong Zhang, Beier Jiang, Ching Chan, Jhong-He Yu, Yu-Pin Lu, KwiMi Chung, Laurent Zimmerli

**Affiliations:** 1 Department of Life Science and Institute of Plant Biology, National Taiwan University, Taipei, Taiwan; 2 Howard Hughes Medical Institute, New York University Langone School of Medicine, New York, NY, USA; 3 Department of Biochemistry and Molecular Pharmacology, New York University Langone School of Medicine, New York, NY, USA; 4 Bioproduction Research Institute, National Institute of Advanced Industrial Science and Technology, Higashi, Tsukuba, Ibaraki, Japan

**Keywords:** *Arabidopsis thaliana*, *Botrytis cinerea*, ethylene response factor, NINJA, pattern-triggered immunity, *Pseudomonas syringae*, transcription factor

## Abstract

Recognition of microbe-associated molecular patterns (MAMPs) derived from invading pathogens by plant pattern recognition receptors (PRRs) initiates a subset of defense responses known as pattern-triggered immunity (PTI). Transcription factors (TFs) orchestrate the onset of PTI through complex signaling networks. Here, we characterized the function of ERF19, a member of the *Arabidopsis thaliana* ethylene response factor (ERF) family. ERF19 was found to act as a negative regulator of PTI against *Botrytis cinerea* and *Pseudomonas syringae*. Notably, overexpression of *ERF19* increased plant susceptibility to these pathogens and repressed MAMP-induced PTI outputs. In contrast, expression of the chimeric dominant repressor *ERF19–SRDX* boosted PTI activation, conferred increased resistance to the fungus *B. cinerea*, and enhanced elf18-triggered immunity against bacteria. Consistent with a negative role for ERF19 in PTI, MAMP-mediated growth inhibition was weakened or augmented in lines overexpressing *ERF19* or expressing *ERF19–SRDX*, respectively. Using biochemical and genetic approaches, we show that the transcriptional co-repressor Novel INteractor of JAZ (NINJA) associates with and represses the function of ERF19. Our work reveals ERF19 as a novel player in the mitigation of PTI, and highlights a potential role for NINJA in fine-tuning ERF19-mediated regulation of Arabidopsis innate immunity.

## Teaser

Phenotypic analyses of a new ethylene response factor, ERF19, involved in the negative regulation o*f* Arabidopsis pattern-triggered immunity.

## Introduction

Plants have adopted sophisticated defense mechanisms to fight off invading pathogens. Initiation of plant defense responses relies on the recognition of non-self organisms. Plants utilize pattern recognition receptors (PRRs) as the first line of surveillance to detect incoming threats posed by pathogens. Plant PRRs perceive microbe-associated molecular patterns (MAMPs), which are molecular structures conserved among microbes and crucial for the survival of microbes (Macho and Zipfel, 2014; Zipfel, 2014). For example, flg22, the active epitope of the bacterial MAMP flagellin, is recognized by the PRR FLAGELLIN SENSING2 (FLS2) (Felix *et al.*, 1999; Gómez-Gómez and Boller, 2000), and the EF-Tu RECEPTOR (EFR) recognizes the conserved peptide elf18 derived from bacterial EF-Tu, which is one of the most abundant proteins in bacteria (Kunze *et al.*, 2004; Zipfel *et al.*, 2006). The fungal MAMP chitin, an important constituent of fungal cell walls (Silipo *et al.*, 2010), is perceived by CHITIN ELICITOR RECEPTOR KINASE1 (CERK1) and LYSM-CONTAINING RECEPTOR-LIKE KINASE 5 (LYK5) (Miya *et al.*, 2007; Wan *et al.*, 2008; Cao *et al.*, 2014). MAMP recognition induces pattern-triggered immunity (PTI), restricting the incursion and proliferation of potential pathogens (Boller and Felix, 2009; Schwessinger and Ronald, 2012; Newman *et al.*, 2013).

Activation of PTI involves massive transcriptional reprogramming to mount defense responses against invading pathogens (Bigeard *et al.*, 2015; Tsuda and Somssich, 2015; Garner *et al.*, 2016; Birkenbihl *et al.*, 2017*a*). General PTI responses include reinforcement of the cell wall through deposition of callose and production of defense-related proteins (Boller and Felix, 2009). Pathogenesis-related (PR) proteins and plant defensins (PDFs) represent two major classes of defense-related proteins with diverse antimicrobial activities (Thomma *et al.*, 2002; van Loon *et al.*, 2006). In Arabidopsis, *PR1* and *PR2* are induced after inoculation with the hemi-biotrophic bacterium *Pseudomonas syringae* pv. *tomato* (*Pst*) DC3000 and are marker genes for flg22 and elf18 treatments (Lu *et al.*, 2009; Choi *et al.*, 2012; Nomura *et al.*, 2012), whereas *PDF1.2* and *PDF1.3*, which are induced by the necrotrophic fungus *Botrytis cinerea*, serve as potential markers for chitin elicitation (Pieterse *et al.*, 2009, 2012; Meng *et al.*, 2013).

Activation of plant immunity requires a high expense of energy, and excessive immune responses reduce plant fitness, hampering plant growth and survival (Bolton, 2009; Katagiri and Tsuda, 2010; Kim *et al.*, 2014). Transcription factors (TFs) lie at the heart of transcriptional reprogramming, and the ethylene response factor (ERF) TF family plays a key role in orchestrating the balance of defense outputs (Nakano *et al.*, 2006; Huang *et al.*, 2016; Jin *et al.*, 2017). Perturbation of key immune regulators may tip the balance and lead to growth retardation. For example, direct activation of ERF6 enhances Arabidopsis resistance to *B. cinerea* and induces constitutive activation of defense genes (Meng *et al.*, 2013). However, these plants exhibit a severe dwarf phenotype, which might be the result of strong defense activation (Meng *et al.*, 2013).

In order to maintain appropriate levels of defense activation, TFs that negatively regulate immunity need to work in concert with defense-activating TFs. For example, the pathogen-induced *ERF4* (*ERF078*) and *ERF9* (*ERF080*) negatively regulate Arabidopsis resistance against fungal pathogens and activation of *PDF1.2* (McGrath *et al.*, 2005; Maruyama *et al.*, 2013). In addition, transcriptional activities of TFs are modulated in a post-translational manner to ensure timely activation or repression of immune signaling cascades (Licausi *et al.*, 2013). Typically, ETHYLENE INSENSITIVE 3 (EIN3) transactivates *ERF1* (*ERF092*), but the transactivation function of EIN3 is repressed in the presence of JASMONATE ZIM-DOMAIN 1 (JAZ1) (Zhu *et al.*, 2011). Notably, JAZ1 interacts with EIN3 and recruits the transcriptional co-repressor Novel Interactor of JAZ (NINJA) with TOPLESS (TPL) or TPL-related proteins (TPRs) (Pauwels *et al.*, 2010; Zhu *et al.*, 2011). EIN3-mediated activation of *ERF1* is de-repressed when JAZ1 is degraded upon accumulation of jasmonic acid (JA) that occurs after pathogen attack (De Vos *et al.*, 2005; Chini *et al.*, 2007; Zhu *et al.*, 2011). JAZ1-imposed repression on EIN3 ensures that *ERF1* and ERF1-targeted defense genes such as *PDF1.2* are not induced in the absence of pathogen invasion (Pieterse *et al.*, 2012).

While there are increasing reports showing that ERFs are involved in plant defense, studies centered on ERFs regulating PTI remain sparse (Bethke *et al.*, 2009; Meng *et al.*, 2013; Xu *et al.*, 2017). Here we report that the pathogen- and MAMP-induced *ERF19* plays a negative role in Arabidopsis immunity against both fungal and bacterial pathogens. Notably, overexpression of *ERF19* or repression of ERF19 function through expression of the chimeric dominant repressor *ERF19–SRDX* leads to decreased and increased PTI responses, respectively. Our data further suggest that ERF19 functions as a modulator in MAMP-mediated growth inhibition and may serve as a buffering mechanism to prevent detrimental effects of excessive PTI. Moreover, our biochemical and genetic approaches showed that NINJA associates with and represses the function of ERF19, suggesting another layer of control over PTI activation. Collectively, our functional studies on ERF19 provide novel evidence about an ERF involved in the regulation of PTI and new insights into the dynamic regulation of plant immunity.

## Materials and methods

### Biological materials and growth conditions

Growth conditions of *Arabidopsis thaliana* (L. Heyhn.) and *Nicotiana benthamiana* were described previously (Yeh *et al.*, 2016). *Arabidopsis* ecotype Col-0 was used as the wild-type (WT) for the experiments unless stated otherwise. We obtained mutants *npr1-1* from X. Dong (Duke University, Durham, NC, USA), *ein2-1* from the Arabidopsis Biological Resource Center (https://abrc.osu.edu/), *coi1-16* (Col-6 background) from J.G. Turner (University of East Anglia, Norwich, UK), and *ninja-1* from E.E. Farmer (University of Lausanne, Switzerland). The Arabidopsis transgenic line 35S:GFP was obtained from K. Wu (National Taiwan University, Taipei, Taiwan). The fungus *B. cinerea* was obtained from C.-Y. Chen (National Taiwan University, Taipei, Taiwan) and was grown on potato dextrose broth (PDB)–agar plates in the growth chamber where Arabidopsis plants were grown (Zimmerli *et al.*, 2001). The bacterium *Pst* DC3000 was provided by B.N. Kunkel (Washington University, St. Louis, MO, USA) and was grown at 28 °C, 200 rpm in King’s B medium with 50 mg l^–1^ rifampicin.

### Preparation of chemicals

Chitin (#C9752, Sigma), and flg22 and elf18 peptides (Biomatik) were suspended in deionized water. β-Estradiol (β-Est, #E2758, Sigma) was prepared in DMSO.

### Pathogen infection assays

Droplet inoculation with *B. cinerea* and assessment of disease symptoms were performed as previously described (Catinot *et al.*, 2015), except that 8 µl of *B. cinerea* inoculum per leaf were used in this study. For spray inoculation with *B. cinerea*, the spore suspension (10^5^ spores ml^–1^ in 1/4 PDB) was evenly sprayed on the leaves of 4-week-old plants until run-off occurred. The infected plants were kept at 100% relative humidity, and disease development was scored at 5 days post-inoculation (dpi). Dip inoculation with *Pst* and assessment of bacterial populations were performed as described (Yeh *et al.*, 2016). To assess PTI-mediated resistance to *Pst*, assays were performed as previously described with slight modifications (Liu *et al.*, 2015). Briefly, five leaves per plant were syringe infiltrated with deionized water or 10 nM elf18 prior to syringe infiltration of 10^6^ cfu ml^–1^*Pst* solution. The inoculated plants were kept at 100% relative humidity overnight. Bacterial titers were determined at 2 dpi as described (Zimmerli *et al.*, 2000).

### Generation of transgenic plants

The coding sequence (CDS) of *ERF19* without a stop codon was amplified from Col-0 cDNA with ERF19-F1 and ERF19-R1 primers and cloned into pCR8-TOPO vector (Invitrogen) to create pCR8-ERF19. The *ERF19* CDS was subcloned into pMDC83 (Curtis and Grossniklaus, 2003) and pEarleyGate103 (Earley *et al.*, 2006) vectors via LR reaction (Thermo Fisher Scientific) to create pMDC83-ERF19 and pEarleyGate103-ERF19 constructs, respectively. To create the inducible construct, the ERF19–green fluorescent protein (GFP) CDS was partially digested from pEarleyGate103-ERF19 with *Xho*I and *Pac*I. The ERF19–GFP fragment was ligated with pMDC7 vector (Zuo *et al.*, 2000; Curtis and Grossniklaus, 2003) digested with the same enzymes to create pMDC7-ERF19. To construct chimeric ERF19–SRDX, the genomic fragment of *ERF19* including its promoter region (base pairs –1 to –1535) was amplified by PCR using ERF19-F2 and ERF19-R2 primers. The product was digested with *Asc*I and *Sma*I, and then introduced into the same enzyme-treated VB0227 vector. The complete *ProERF19:ERF19-SRDX:HSP* part was transferred into the pBCKH(VB0047) (Mitsuda *et al.*, 2011) binary vector by LR reaction to create pBCKH-ERF19-SRDX. *Agrobacterium tumefaciens* GV3101 was used to deliver the constructs into plants (Martinez-Trujillo *et al.*, 2004). Constructs pMDC83-ERF19, pEarleyGate103-ERF19, pMDC7-ERF19, and pBCKH-ERF19-SRDX were used to generate transgenic ERF19-OE, ERF19-OE/*ninja-1*, ERF19-iOE, and ERF19–SRDX lines, respectively. Independent homozygous T_3_ lines with a single T-DNA insertion were used for the experiments. All primers used in this study are summarized in Supplementary Table S1 at *JXB* online.

### Treatment with β-Est

Twelve-day-old seedlings and 5-week-old plants were treated with 20 µM β-Est by submergence in liquid half-strength Murashige and Skoog (1/2 MS) and syringe infiltration, respectively, 24 h before downstream experiments.

### Subcellular localization

Β-Est-treated, 12-day-old ERF19-iOE1 and 35S:GFP seedlings were vacuum infiltrated with DAPI solution (5 µg ml^–1^) for 2 min and washed three times with distilled water. The GFP and DAPI signals in the roots were imaged with a Zeiss LSM 780 confocal microscope.

### RT–PCR

To monitor MAMP- or pathogen-induced *ERF19*, 12-day-old seedlings were incubated in liquid 1/2 MS for one night before treatments with 200 µg ml^–1^ chitin, 100 nM flg22, 100 nM elf18, 5 × 10^5^*B. cinerea* spores ml^–1^, or 10^7^ cfu ml^–1^*Pst*. To prepare the microbial inoculants, *B. cinerea* spores and *Pst* were pelleted by centrifugation at 3000 *g* for 5 min and resuspended in 1/2 MS. Total RNA isolation, reverse transcription, and real-time PCR (RT–PCR) analyses were performed as described (Catinot *et al.*, 2015). The gene *UBIQUITIN 10* (*UBQ10*) was used for normalization. For RT–PCR, 2 µl of cDNA were used as template, and standard PCR conditions were applied as described (Huang *et al.*, 2014). *UBQ10* was used as a loading control.

### Callose deposition assays

Fourteen-day-old seedlings were incubated in liquid 1/2 MS for one night before treatments with 200 µg ml^–1^ chitin, 100 nM flg22, 100 nM elf18, or deionized water. Twenty-four hours later, callose deposits were stained and quantified as previously described (Kohari *et al.*, 2016; Yeh *et al.*, 2016).

### Protoplast preparation and transfection

Arabidopsis protoplasts were prepared from the leaves of 5-week-old plants as previously described (Wu *et al.*, 2009). Polyethylene glycol-mediated protoplast transfection was performed as described (Yoo *et al.*, 2007).

### Protoplast transactivation (PTA) assays

PTA assays were performed as previously described (Hsieh *et al.*, 2013). The reporter plasmid consists of the gene encoding firefly luciferase (fLUC) under the control of upstream activation sequence (UAS) targeted by the yeast GAL4 TF. The reference plasmid carries the gene encoding Renilla luciferase (rLUC) under the control of the 35S promoter. Effector plasmid harboring the DNA-binding domain of GAL4 expressed from the 35S promoter was used as the empty vector control (GAL4DB). The fragment of *ERF19* CDS amplified by PCR using ERF19-F3 and ERF19-R3 primers was digested with *Xma*I and *Sal*I, and then introduced into GAL4DB to create GAL4DB-ERF19 effector plasmid. To construct GAL4DB-ERF19-SRDX effector plasmid, the fragment ERF19–SRDX, amplified from pBCKH-ERF19-SRDX with primers ERF19-F and SRDX-R, was ligated with the vector backbone, amplified from GAL4DB with primers pGAL4-F and pGAL4-R, by blunt-end cloning. To create the NINJA, HDA6, and HDA19 effector plasmids, the CDS of *NINJA*, *HDA6*, and *HDA19* were amplified from Col-0 cDNA, introduced into pCR8-TOPO vector, and subcloned into pGWHA, a plasmid modified from p2FGW7 (Karimi *et al.*, 2002), by substituting the GFP tag with a single HA tag, by LR reaction. The effector plasmids, reporter plasmids, and reference plasmids were transfected to Arabidopsis protoplasts at the ratio of 5:4:1. After 24 h, the luciferase activities were analyzed using the Dual-Luciferase Reporter Assay System (Promega). Data are presented as the normalized fLUC activities relative to the no effector control (set as 1).

### MAMP-induced growth inhibition

Growth inhibition experiments were performed as described (Ranf *et al.*, 2011). Briefly, ten 5-day-old seedlings of the same genotype were transferred into 6-well plates supplemented with liquid 1/2 MS (0.5 g l^–1^ MES, 0.25% sucrose, pH 5.7). The seedlings were treated with water or MAMPs at the indicated concentration. The treated seedlings were further grown for another 10 d under normal growth conditions. Ten seedlings in a single well were blotted dry on tissue paper and weighed as a whole.

### Co-immunoprecipitation (Co-IP) assay in Arabidopsis protoplasts

Full-length CDS of *NINJA*, *HDA6*, and *HDA19* were cloned into pCR8-TOPO entry vector, and subcloned into pEarleyGate103 (Earley *et al.*, 2006) by LR reaction. The GFP empty vector control was created by digesting pEarleyGate103 with *Xho*I to remove the Gateway cassette. The *ERF19* CDS from pCR8-ERF19 was first introduced into pGWB14 vector with a C-terminal triple HA fusion (Nakagawa *et al.*, 2007) via LR reaction, and the fragment 35S:ERF19-HA_3_:NOS was amplified with 35S-F and NOS-R primers by PCR. This fragment was cloned into pCR8-TOPO to create a plant expression plasmid with high copy number. Protoplast transfection and Co-IP were performed as previously described (Yeh *et al.*, 2015).

### Bimolecular fluorescence complementation (BiFC) in *N. benthamiana*

Using LR reaction, the CDS of *ERF19*, *NINJA*, *HDA6*, and *HDA19* were introduced into BiFC vectors carrying split yellow fluorescent protein (YFP) fragments (Waadt *et al.*, 2008). To create the construct for the nuclear marker, the nuclear localization signal (NLS) was fused to the N-terminus of mCherry by PCR with primers NLS-mCherry-F and mCherry-R. This fragment was cloned into pENTR/D-TOPO vector and digested with *Sma*I to create a blunt-end vector. The vector was then ligated with the PCR fragment mCherry–NLS, amplified with primers mCherry-F and mCherry-NLS-R, to create the complete NLS–mCherry–mCherry–NLS sequence. This sequence was introduced into pEarleyGate100 (Earley *et al.*, 2006) by LR reaction to create the construct for the nuclear marker. The constructs were transformed into *A. tumefaciens* GV3101 by electroporation. Transient expression in *Nicotiana benthamaian* was performed as described (Roux *et al.*, 2011), except that the *Agrobacterium* strains carrying the BiFC constructs were mixed 1:1 to a final OD_600_ of 0.4 for each strain, and the nuclear marker strain was added to a final OD_600_ of 0.1. Two days later, the transiently expressing leaves were imaged with a Zeiss LSM 780 confocal microscope.

### Protein extraction in Arabidopsis seedlings

Extraction of total proteins from Arabidopsis seedlings was performed as previously described (Tsugama *et al.*, 2011).

### Immunoblotting

Immunoblotting was performed as previously described (Yeh *et al.*, 2016). The primary antibodies used in this study were anti-GFP (#sc-9996, Santa Cruz Biotechnology) and anti-HA (#sc-7392, Santa Cruz Biotechnology).

### Yeast two-hybrid (Y2H) assays

Using LR reaction, the full-length CDS of *ERF19* was subcloned into the pGADT7 vector, and *NINJA*, *HDA6*, and *HDA19* CDS were introduced into the pGBKT7 vector. The constructs were transformed into yeast strain AH109 based on the LiAc-mediated transformation protocol following the manufacturer’s instructions (Clontech). At least 10 co-transformed yeast colonies were plated on Synthetic Drop-Out (SD) medium supplemented with X-α-Gal (Clontech) but without leucine, tryptophan, and histidine (-L-W-H). The plates were incubated at 30 °C for 3 d to test the nutritional marker gene expression and galactosidase activity of the MEL1 reporter protein.

### Accession numbers

Sequence data from this article can be found in the Arabidopsis Genome Initiative under the accession numbers: *ERF19* (AT1G22810), *NINJA* (AT4G28910), *HDA6* (AT5G63110), *HDA19* (AT4G38130), *UBQ10* (AT4G05320), *PDF1.2* (AT5G44420), *PDF1.3* (AT2G26010), *PR1* (AT2G14610), and *PR2* (AT3G57260).

## Results

### Overexpression of *ERF19* enhances Arabidopsis susceptibility to pathogens

**Fig. 1.**
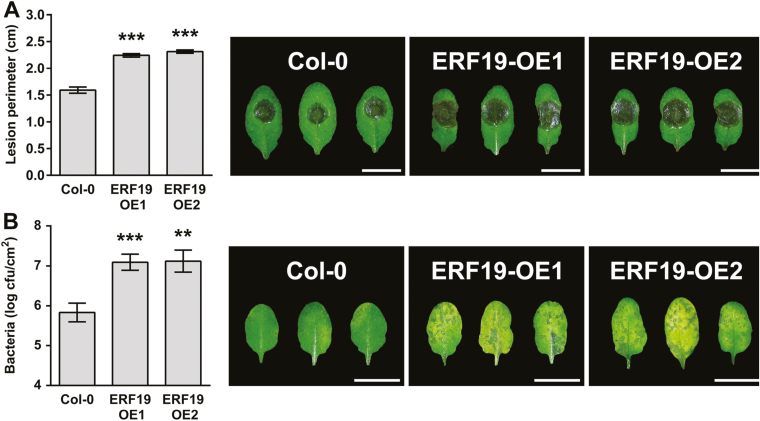
ERF19-OEs are hypersusceptible to *B. cinerea* and *Pst* DC3000. (A) *B. cinerea*-mediated lesions. Leaves of 5-week-old ERF19-OEs were droplet inoculated with 8 µl of *B. cinerea* spore suspension (10^5^ spores ml^–1^ in 1/4 PDB). Disease symptoms were photographed and lesion perimeters were measured at 3 days post-inoculation (dpi). Data represent the average ±SE of at least 72 lesion perimeters pooled from three independent experiments each with at least six plants per line. Asterisks indicate a significant difference from Col-0 based on a *t*-test (***P<0.001). (B) *Pst* growth and symptoms. Five-week-old plants were dip inoculated with 10^6^ cfu ml^–1^*Pst*, and symptoms were photographed at 3 dpi. Bacterial populations in the leaves were evaluated at 2 dpi. Values represent the average ±SE from three independent experiments pooled, each with five plants per line (*n*=15). Asterisks indicate a significant difference from Col-0 based on a *t*-test (**P<0.01; ***P<0.001).

To identify TFs involved in the regulation of Arabidopsis defenses against the fungal pathogen *B. cinerea*, we designed a screen to evaluate the resistance of Arabidopsis from the *At*TORF-Ex collection (Weiste *et al.*, 2007; Wehner *et al.*, 2011) to this pathogen. Notably, we found a transgenic line overexpressing *ERF19/ERF019* (At1g22810, HA-ERF19) that developed increased disease lesions after drop inoculation with *B. cinerea* spores (Supplementary Fig. S1A–C). To confirm that the increased susceptibility phenotype of the HA-ERF19 line to *B. cinerea* was not due to multiple transformation events (Weiste *et al.*, 2007), we generated additional Arabidopsis lines expressing the CDS of *ERF19* fused with *GFP* under the control of the *Cauliflower mosaic virus* 35S (CaMV 35S) promoter in the Col-0 background. Two independent lines (ERF19-OE1 and -OE2), expressing high levels of *ERF19* mRNA and ERF19–GFP proteins (Supplementary Fig. S2A, B), were selected for further analyses. Confirming the increased susceptibility to *B. cinerea* observed in HA-ERF19 (Supplementary Fig. S1B, C), ERF19-OE1 and -OE2 developed larger disease lesions than Col-0 after *B. cinerea* drop inoculation (Fig. 1A). In addition to ERF19-OEs, we generated transgenic lines expressing the CDS of the *ERF19-GFP* fusion under the control of the β-Est-inducible XVE system (ERF19-iOEs). Overexpression of *ERF19* and ERF19–GFP was β-Est dependent (Supplementary Fig. S2C, D). Confirming data observed in lines constitutively overexpressing *ERF19* (Fig. 1A), increased susceptibility to *B. cinerea* was observed in ERF19-iOEs treated with β-Est, but not in mock controls treated with DMSO (Supplementary Fig. S3). Importantly, β-Est treatment did not alter Col-0 resistance to *B. cinerea* as compared with the DMSO-treated control (Supplementary Fig. S3), indicating that the increased susceptibility to *B. cinerea* in ERF19-iOEs is specifically linked to overexpression of *ERF19* rather than to the β-Est treatment. In summary, our phenotypic analyses on HA-ERF19, ERF19-OEs, and ERF19-iOEs show that overexpression of *ERF19* enhances Arabidopsis susceptibility to *B. cinerea*. Confirming earlier work (Scarpeci *et al.*, 2017), the rosette leaves of 5-week-old ERF19-OEs exhibited different degrees of inward curling, and the rosette biomass of ERF19-OEs was smaller than that of the WT Col-0 (Supplementary Fig. S4A, B). However, unlike ERF19-OEs, the rosettes of ERF19-iOE and Col-0 plants were indistinguishable when grown in laboratory conditions (Supplementary Fig. S4C). Since ERF19-OE and β-Est-treated ERF19-iOE lines showed similar enhanced susceptibility to *B. cinerea*, the observed enhanced susceptibility phenotype to *B. cinerea* in ERF19-OEs (Fig. 1A) is probably not linked to the altered growth phenotype of these OE lines.

To dissect the role of ERF19 in Arabidopsis resistance to microbial pathogens further, ERF19-OEs and Col-0 plants were dip inoculated with *Pst* DC3000, and disease symptoms were evaluated 3 d later. ERF19-OEs developed increased disease symptoms as indicated by widespread chloroses on the leaves of ERF19-OEs (Fig. 1B). Consistently, bacterial growth assays revealed that ERF19-OEs harbored at least 10 times more bacteria than Col-0 plants (Fig. 1B), indicating that ERF19-OEs were hypersusceptible to *Pst* bacteria. Collectively, these data suggest that overexpression of *ERF19* in Arabidopsis induces hypersusceptibility to both fungal and bacterial pathogens.

### *ERF19* is transiently induced by MAMPs

**Fig. 2.**
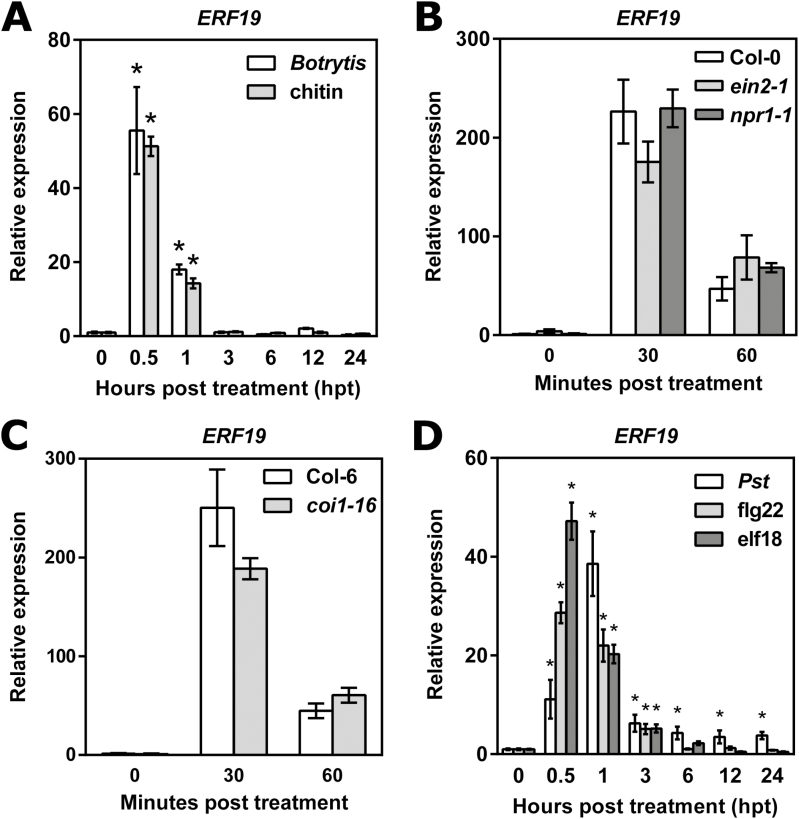
Expression analyses of *ERF19*. (A) Time course expression of *ERF19* after inoculation with *B. cinerea* or treatment with chitin. Twelve-day-old seedlings were inoculated with a suspension of 5 × 10^5^*B. cinerea* spores ml^–1^ or treated with 200 µg ml^–1^ chitin. Samples were collected at the indicated time points, and *ERF19* expression was determined by qRT-PCR. After normalization with *UBQ10*, *ERF19* expression levels were compared with time 0 (defined value of 1). Data represent the mean ±SD of three replicates (*n*=3). Asterisks denote values significantly different from time 0 based on a *t*-test (*P<0.05). (B) Chitin-induced *ERF19* in *ein2-1*, *npr1-1*, and WT Col-0. Twelve-day-old seedlings were treated with 200 µg ml^–1^ chitin for 30 min and 60 min. Samples were collected at the indicated time points, and *ERF19* expression was analyzed as in (A). Data represent the mean ±SD of four replicates (*n*=4). No significant differences in *ERF19* expression were found between Col-0 and the mutants at different time points (*t*-test; *P*>0.05). (C) Chitin-induced *ERF19* expression in *coi1-16* mutant and its WT Col-6 was evaluated as in (B). Data represent the mean ±SD of four replicates (*n*=4). No significant differences of *ERF19* expression were found between Col-6 and the *coi1-16* mutant at different time points (*t*-test; *P*>0.05). (D) Time course expression of *ERF19* after inoculation with *Pst* or after treatment with flg22 or elf18. Twelve-day-old seedlings were inoculated with 10^7^ cfu ml^–1^*Pst*, or treated with 100 nM flg22 or 100 nM elf18, and samples were collected at the indicated time points. Analysis of *ERF19* expression was performed and presented as in (A). Asterisks denote values significantly different from the respective time 0 based on a *t*-test (*P<0.05).

To evaluate further the role of ERF19 in Arabidopsis immunity, we first monitored the expression of *ERF19* in Col-0 seedlings after inoculation with *B. cinerea* spores or treatment with the fungal MAMP chitin over a 24 h period. *ERF19* transcripts were up-regulated by *B. cinerea* spores or chitin within half an hour, and steadily declined at later time points (Fig. 2A). These results are consistent with previous reports showing that *ERF19* is rapidly induced by chitin and chitin derivatives (Ramonell *et al.*, 2005; Libault *et al.*, 2007; Fakih *et al.*, 2016). Signaling pathways of phytohormones such as salicylic acid (SA), JA, and ethylene (ET) are important for transcriptional regulation of immune regulators (Pieterse *et al.*, 2009, 2012). To dissect the regulation of chitin-induced *ERF19*, we examined the expression of *ERF19* after chitin treatment in *npr1-1*, *coi1-16*, and *ein2-1* mutants, which are defective in SA, JA, and ET signaling pathways, respectively (Guzmán and Ecker, 1990; Cao *et al.*, 1994; Ellis and Turner, 2002). Chitin-induced *ERF19* transcripts in *ein2-1*, *npr1-1*, and *coi1-16* were similar to their respective WT within 1 h post-treatment (Fig. 2B, C). These data indicate that rapid induction of *ERF19* by chitin is unaffected when SA, JA, or ET signaling is impaired.

Since overexpression of *ERF19* induced hypersusceptibility to *Pst* bacteria, we also monitored the expression of *ERF19* in Col-0 seedlings after inoculation with *Pst*, or after treatment with the bacterial MAMPs flg22 or elf18. Similarly to *B. cinerea* spores or chitin, inoculation with *Pst* or treatments with flg22 or elf18 transiently up-regulated *ERF19* for 1 h, but *ERF19* transcripts declined steadily afterwards (Fig. 2D). To ensure that *ERF19* expression levels observed in Fig. 2A, D are not a consequence of the experimental conditions, we also performed a time course study of *ERF19* expression after mock (water or 1/2 MS) treatment. No up-regulation of ERF19 was observed in the mock controls (Supplementary Fig. S5). Together these data show that ERF19 is transiently up-regulated upon activation of Arabidopsis immunity.

### PTI responses are down-regulated in *ERF19* overexpression lines

**Fig. 3.**
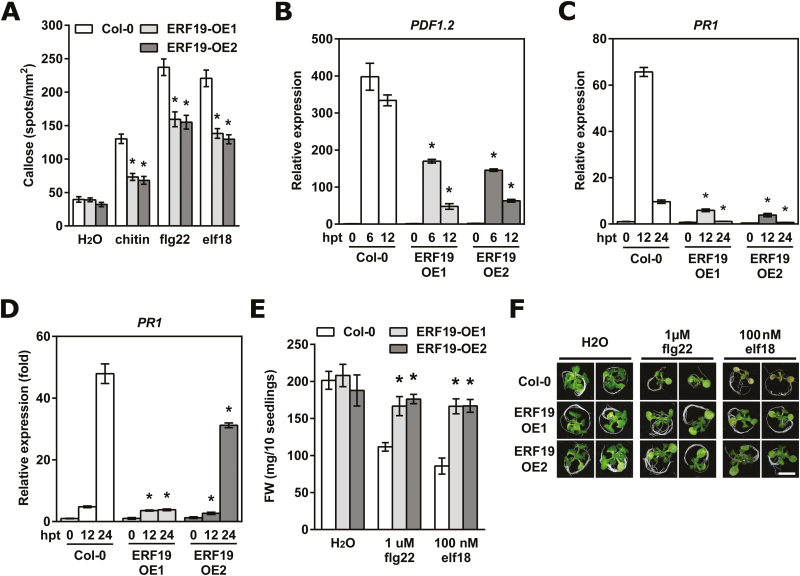
ERF19 is involved in PTI. (A) MAMP-induced callose deposition in ERF19-OEs. Fourteen-day-old seedlings were treated with deionized water (mock control), 200 µg ml^–1^ chitin, 100 nM flg22, or 100 nM elf18, and samples were collected 24 h later for aniline blue staining. Data represent the average numbers of callose deposits per square millimeter ±SE pooled from four independent experiments each with at least six biological repeats (*n* >24). Asterisks denote values significantly different from the respective Col-0 controls based on a *t*-test. (*P<0.01). (B–D) Activation of PTI marker genes in ERF19-OEs. Chitin-induced *PDF1.2* (B), flg22-induced *PR1* (C), and elf18-induced *PR1* (D) in ERF19-OEs were determined by qRT-PCR. Twelve-day-old seedlings were treated with 200 µg ml^–1^ chitin, 1 µM flg22, or 1 µM elf18. Samples were collected at the indicated time points. After normalization with *UBQ10*, expression levels of PTI marker genes were compared with Col-0 at time 0 (defined value of 1). Data represent the mean ±SD of three replicates (*n*=3). Asterisks denote values significantly different from the respective Col-0 controls based on a *t*-test (*P<0.05). (E) MAMP-mediated growth inhibition in ERF19-OEs. Five-day-old seedlings were grown in liquid 1/2 MS supplemented with 1 µM flg22 or 100 nM elf18. Seedlings were weighed 10 d after treatment. Data represent the average fresh weight of 10 seedlings ±SE from three independent experiments (*n*=3). Asterisks indicate a significant difference from the respective Col-0 controls based on a *t*-test (*P<0.05). (F) Representative seedlings treated as in (E).

Plants utilize PTI as a defense mechanism to ward off diverse pathogens (Boller and Felix, 2009; Huang and Zimmerli, 2014), and perturbation of PTI compromises plant defense against both fungal and bacterial pathogens (Tsuda *et al.*, 2009; Kim *et al.*, 2014). Since ERF19-OEs showed an increased susceptibility to both *B. cinerea* and *Pst* DC3000 and since *ERF19* was up-regulated by fungal and bacterial MAMPs (Fig. 2A, B), we evaluated whether ERF19 is involved in PTI. Towards this goal, we first measured callose deposition, a PTI output activated by fungal and bacterial MAMPs (Millet *et al.*, 2010; Shinya *et al.*, 2014), in ERF19-OEs and Col-0. While the water-treated callose deposits were similar between ERF19-OEs and Col-0, callose deposition induced by chitin, flg22, or elf18 was significantly impaired in ERF19-OEs (Fig. 3A). Next, the expression of PTI maker genes was monitored in ERF19-OEs and Col-0 after MAMP treatments. Transcripts of chitin-induced *PDF1.2* and *PDF1.3*, as well as flg22- or elf18-induced *PR1* and *PR2* were lower in ERF19-OEs than in Col-0 (Fig. 3B–D; Supplementary Fig. S6A–C), indicating a defective up-regulation of these PTI marker genes when *ERF19* is overexpressed. Lastly, we tested the plant sensitivities toward flg22- and elf18-mediated growth arrest, a well-documented feature of PTI (Gómez-Gómez and Boller, 2000; Zipfel *et al.*, 2006; Ranf *et al.*, 2011). While treatment with flg22 or elf18 profoundly inhibited the growth of Col-0 seedlings, the MAMP-mediated growth inhibition effect was significantly lower in ERF19-OEs (Fig. 3E, F). Interestingly, smaller sizes of adult ERF19-OEs were not observed at an early developmental stage (compare Fig. 3E, F and Supplementary Fig. S4A, B). Taken together, these results show that overexpression of *ERF19* alters the activation of common PTI responses and MAMP-mediated growth inhibition.

### Expression of the dominant-negative *ERF19–SRDX* transgene enhances Arabidopsis PTI responses

**Fig. 4.**
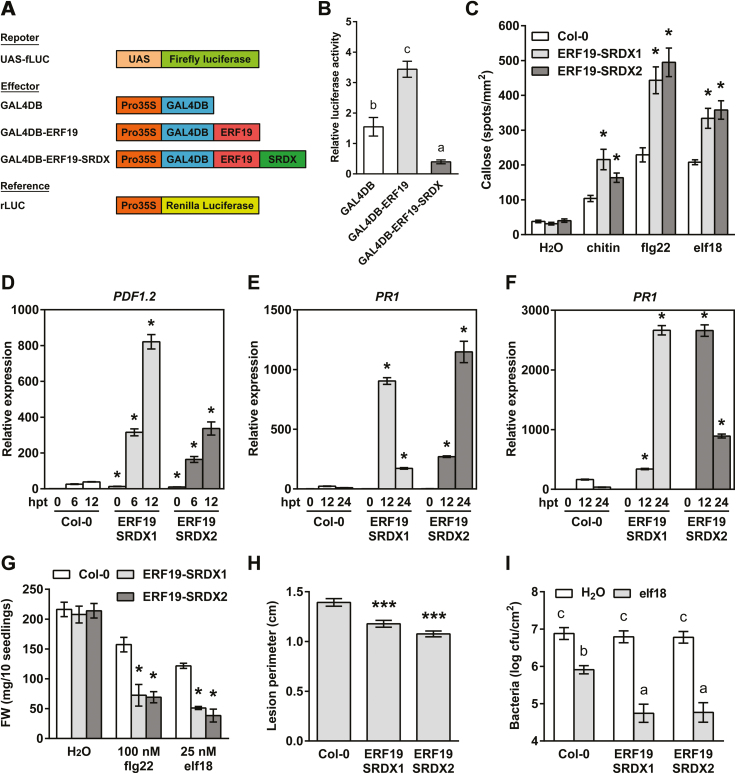
Expression of the dominant repressor *ERF19–SRDX* enhances PTI. (A) Schematic diagrams of reporter, effector, and reference plasmids used in the PTA assay. (B) PTA assay. Relative luciferase activities were evaluated in Arabidopsis protoplasts co-transfected with the reporter plasmid (UAS:fLUC), the effector plasmids (35S:GAL4DB, 35S:GAL4DB-ERF19, or 35S:GAL4DB-ERF19-SRDX), and a calibrator plasmid encoding *rLUC*. Protoplasts transfected without the effector plasmids were used as a control (no effector). All the values were normalized to the rLUC activity and were relative to the values of the no effector control. Values are means ±SE of four independent experiments (*n*=4). Different letters denote significant differences between groups based on a one-way ANOVA (*P*<0.01). (C) MAMP-induced callose deposition in ERF19–SRDXs. Fourteen-day-old seedlings were treated with deionized water (mock control), 200 µg ml^–1^ chitin, 100 nM flg22, or 100 nM elf18, and samples were collected 24 h later for aniline blue staining. Data represent the average numbers of callose deposits per square millimeter ±SE pooled from three independent experiments each with at least six biological repeats (*n* >24). Asterisks denote values significantly different from the respective Col-0 controls based on a *t*-test (*P<0.01). (D–F) Activation of PTI marker genes in ERF19–SRDXs. Chitin-induced *PDF1.2* (D), flg22-induced *PR1* (E), and elf18-induced *PR1* (F) in ERF19–SRDXs were determined by qRT-PCR. Twelve-day-old seedlings were treated with 200 µg ml^–1^ chitin, 1 µM flg22, or 1 µM elf18. Samples were collected at the indicated time points, and *UBQ10* was used for normalization. Relative gene expression levels were compared with Col-0 at time 0 (defined value of 1). Data represent the mean ±SD of three replicates (*n*=3). Asterisks denote values significantly different from the respective Col-0 controls based on a *t*-test (*P<0.05). (G) MAMP-mediated growth inhibition in ERF19–SRDXs. Five-day-old seedlings were grown in liquid 1/2 MS supplemented with 100 nM flg22 or 25 nM elf18. Seedlings were weighed 10 d after treatment. Data represent the average fresh weight (FW) of 10 seedlings ±SE from three independent experiments (*n*=3). Asterisks indicate a significant difference from the respective Col-0 controls based on a *t*-test (*P<0.05). (H) *B. cinerea*-mediated lesions in ERF19–SRDXs. Leaves of 5-week-old plants were droplet inoculated with 8 µl of *B. cinerea* spores (10^5^ spores ml^–1^ in 1/4 PDB). Lesion perimeters were measured at 3 dpi. Data represent the average ±SE of 138 lesion perimeters (*n*=138) pooled from four independent experiments each with at least six plants per line. Asterisks indicate a significant difference from Col-0 based on a *t*-test (***P<0.001). (I) *Pst* DC3000 growth in ERF19–SRDXs. Five-week-old plants were syringe infiltrated with H_2_O or 10 nM elf18 6 h before syringe infiltration with 10^6^ cfu ml^–1^*Pst*. Bacterial populations in the leaves were evaluated at 2 dpi. Values represent the average ±SE from three independent experiments each with three plants per line pooled (*n*=9). Different letters denote significant differences between groups based on a two-way ANOVA (*P*<0.01).

To determine further the biological role of *ERF19*, we aimed to investigate the dominant-negative actions of ERF19 *in planta*, a commonly used strategy for studying TF functions (Mitsuda and Ohme-Takagi, 2009). We first examined the transcriptional activity of ERF19 by using PTA assays based on the GAL4/UAS and dual-luciferase reporter system. In Arabidopsis protoplasts, expression of ERF19 fused to the GAL4DB showed higher luciferase activity than expression of GAL4DB alone (Fig. 4A, B), suggesting that ERF19 acts as a transcription activator. Importantly, PTA assays revealed that the fusion of a plant-specific EAR-motif repression domain (SRDX) (Hiratsu *et al.*, 2003; Mitsuda *et al.*, 2011) to ERF19 successfully converted the activator feature of ERF19 into a repressor (Fig. 4A, B), indicating that the chimeric repressor ERF19–SRDX is appropriate for studying the dominant-negative actions of ERF19.

To assess further the biological function of ERF19–SRDX, the *ERF19* genomic sequence, consisting of the intergenic promoter region (base pairs –1 to –1535), the 5'-untranslated region, and the CDS of *ERF19* fused to the *SRDX* CDS, was expressed in Col-0 to generate ERF19–SRDX lines. The use of a native promoter of *ERF19* better reflects the biological function of ERF19–SRDX than a constitutive promoter (Mitsuda and Ohme-Takagi, 2009). Two independent lines of ERF19–SRDX, of which the transgene *ERF19–SRDX* was chitin responsive, were selected for further analyses (Supplementary Fig. S7A). Unlike ERF19-OEs, the rosettes of ERF19–SRDXs were indistinguishable from those of the Col-0 WT (Supplementary Fig. S7B). To confirm the role of ERF19 in PTI and pathogen resistance, we first analyzed MAMP responses of ERF19–SRDX lines. Remarkably, MAMP-induced callose deposits were higher in ERF19–SRDXs than in Col-0 (Fig. 4C). Similarly, chitin-induced *PDF1.2* and *PDF1.3*, flg22-induced *PR1*, and elf18-induced *PR1* and *PR2*, were higher in ERF19–SRDXs than in Col-0 plants (Fig. 4D–F; Supplementary Fig. S7C, E). Surprisingly, despite enhanced expression of flg22-induced *PR1*, ERF19–SRDXs showed WT expression levels of flg22-induced *PR2* (Supplementary Fig. S7D). Confirming the augmented PTI responses, MAMP-induced growth arrest was much more severe in ERF19–SRDXs (Fig. 4G). Together, these results suggest that transgenic expression of *ERF19–SRDX* enhances Arabidopsis PTI responses and MAMP-induced inhibition of growth.

In ERF19-OEs, the enhanced susceptibility to fungal and bacterial pathogens was correlated with reduced PTI responses. We thus hypothesized that the heightened PTI activation in ERF19–SRDX plants will confer pathogen resistance. As expected, ERF19–SRDXs exhibited smaller disease lesions than Col-0 WT upon *B. cinerea* infection (Fig. 4H), indicating that ERF19–SRDXs were more resistant to *B. cinerea* than Col-0 plants. However, Col-0 and ERF19–SRDXs developed similar *Pst*-mediated disease symptoms (Supplementary Fig. S7F). To highlight the role of ERF19–SRDX in PTI-mediated defense against *Pst* DC3000, we activated Arabidopsis PTI by treatment with 10 nM of the MAMP elf18 prior to *Pst* DC3000 inoculation. In water-treated controls, bacterial growth was similar in Col-0 and ERF19–SRDXs (Fig. 4I), confirming our previous observation showing that ERF19–SRDXs infected with *Pst* DC3000 exhibit WT disease symptoms (Supplementary Fig. S7F). Strikingly, a decrease of *Pst* DC3000 growth by elf18 pre-treatment was significantly stronger in ERF19–SRDXs than in Col-0 plants (Fig. 4I), suggesting that elf18-induced resistance to *Pst* DC3000 was enhanced in ERF19–SRDXs. Together, these results show that the expression of the dominant repressor *ERF19–SRDX* boosts PTI responses and, consequently, can confer increased resistance to fungal and bacterial pathogens. In summary, our phenotypic analyses on ERF19-OEs and ERF19–SRDXs provide genetic evidence that ERF19 plays a negative role in the regulation of Arabidopsis PTI and defense towards pathogens.

### ERF19 is a nuclear TF

**Fig. 5.**
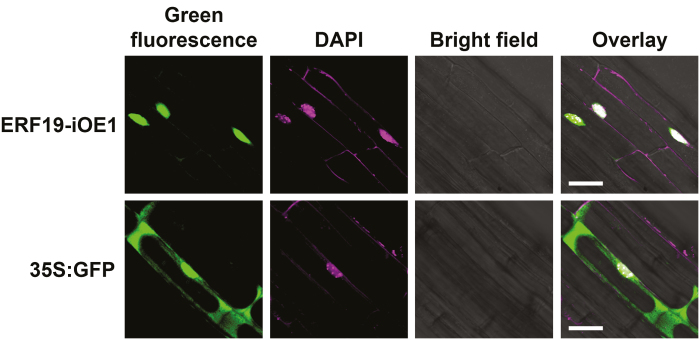
Subcellular localization of ERF19–GFP. Pictures were taken from seedlings of 12-day-old ERF19-iOE1 treated with 20 µM β-Est for 24 h and 35S:GFP transgenic lines. DAPI staining was used to determine the position of nuclei. Strong green fluorescence (green) of ERF19–GFP was co-localized with the DAPI-stained (magenta) nuclei. The scale bar represents 5 µm.

To determine the subcellular localization of ERF19, we took advantage of the high expression levels of ERF19–GFP in β-Est-treated ERF19-iOE1 (Supplementary Fig. S2C). Confocal microscope images revealed that strong GFP signals co-localized with DAPI-stained nuclei in the seedling roots of β-Est-treated ERF19-iOE1 (Fig. 5), indicating that ERF19–GFP is enriched in the nucleus. In contrast, the GFP alone control roots of transgenic seedlings showed a dispersed nuclear and cytoplasmic fluorescence (Fig. 5). These data suggest a nuclear localization for ERF19.

### NINJA associates with and represses ERF19

**Fig. 6.**
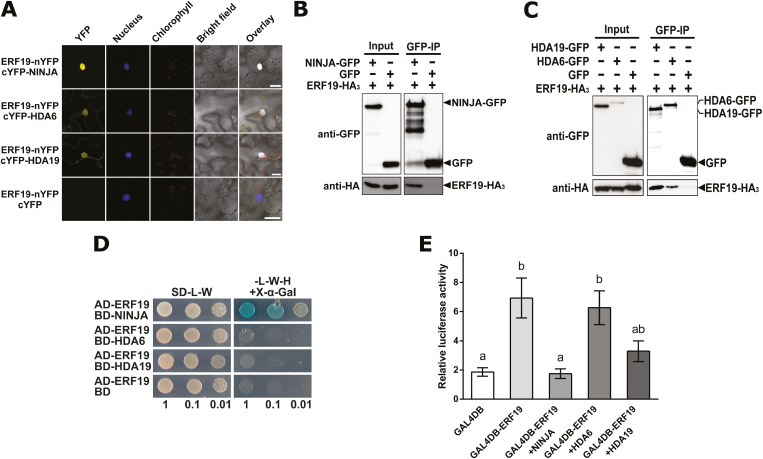
NINJA associates with and represses the transcriptional activity of ERF19. (A) BiFC analysis. *N. benthamiana* plants were co-transformed with the indicated split YFP constructs and a nuclear marker construct carrying NLS–mCherry–mCherry–NLS. YFP fluorescence (yellow), nucleus (blue), chlorophyll autofluorescence (red), bright field, and overlay images are shown. This experiment was performed at least three times with similar results. Scale bars represent 20 µm. (B and C) Analysis of ERF19–NINJA, ERF19–HDA6, and ERF19–HDA19 association by Co-IP. Total proteins from protoplasts expressing GFP, NINJA–GFP, HDA6–GFP, or HDA19–GFP with ERF19-HA_3_ were immunoprecipitated (IP) with anti-GFP antibodies. Total proteins before (input) and after IP (GFP-IP) were immunoblotted with anti-GFP and anti-HA antibodies. Similar results were obtained from three independent experiments. (D) Analysis of ERF19–NINJA, ERF19–HDA6, and ERF19–HDA19 association by Y2H assays. Ten-fold serial dilutions of yeasts expressing the indicated protein fusion to the activation domain (AD) or binding domain (BD) of GAL4 were plated on control (-L-W) or selective (-L-W-H/+X-α-Gal) SD media. Growth and blue staining of the colonies on selective SD medium indicate association between the two fusion proteins. The experiment was performed three times with similar results. (E) PTA assay. Relative luciferase activities of Arabidopsis protoplasts co-transfected with the reporter plasmid (UAS:fLUC), the effector plasmids (35S:GAL4DB, 35S:GAL4DB-ERF19, or 35S:GAL4DB-ERF19 with 35S:NINJA, 35S:HDA6, or HDA19), and a normalization plasmid encoding rLUC. All the values were normalized to the rLUC activity and were relative to the values of the no effector control. Different letters denote significant differences between groups based on a one-way ANOVA (*P*<0.05).

The activities of TFs can be regulated via protein–protein interactions. The identification of TF-interacting proteins is thus crucial to unravel the regulation of TF regulatory networks (Licausi *et al.*, 2013). ERFs were reported to form complexes with co-repressors and histone deacetylases (HDAs) (Kagale and Rozwadowski, 2011). We thus tested whether ERF19 associates with the well-studied HDA6 and HDA19 (Liu *et al.*, 2014) via BiFC assays. Since HDA6 and HDA19 are components of the NINJA co-repressor complex (Zhang *et al.*, 2017), we also included NINJA in the assay. Reconstitution of YFP in the nucleus was observed when ERF19 fused to the N-terminus of YFP was co-expressed with NINJA, HDA6, or HDA19 fused to the C-terminus of YFP in the leaves of *N. benthamiana* (Fig. 6A), suggesting that ERF19 can interact with these proteins *in planta*. Co-expression of ERF19–nYFP and cYFP alone did not show any yellow fluorescence (Fig. 6A). Consistently, Co-IP analyses revealed that ERF19-HA_3_ proteins could be pulled down along with NINJA–GFP, HDA6–GFP, and HDA19–GFP proteins, but not GFP alone (Fig. 6B, C), further strengthening the idea that ERF19 associates with these proteins *in planta*. However, in our Y2H assays, only NINJA is capable of associating with ERF19 *in vitro* (Fig. 6D), suggesting that ERF19–HDA6 and ERF19–HDA19 association requires plant-specific factors. As NINJA, HDA6, and HDA19 are probably part of a co-repressor complex, we tested via PTA analysis whether NINJA, HDA6, or HDA19 alters the transcriptional activity of ERF19. Interestingly, only co-transfection of NINJA with GAL4DB-ERF19 strongly and significantly repressed ERF19-activated luciferase activity (Fig. 6E). Taken together, these data suggest that NINJA associates with ERF19 and plays a negative role in the transcriptional activity of ERF19.

**Fig. 7.**
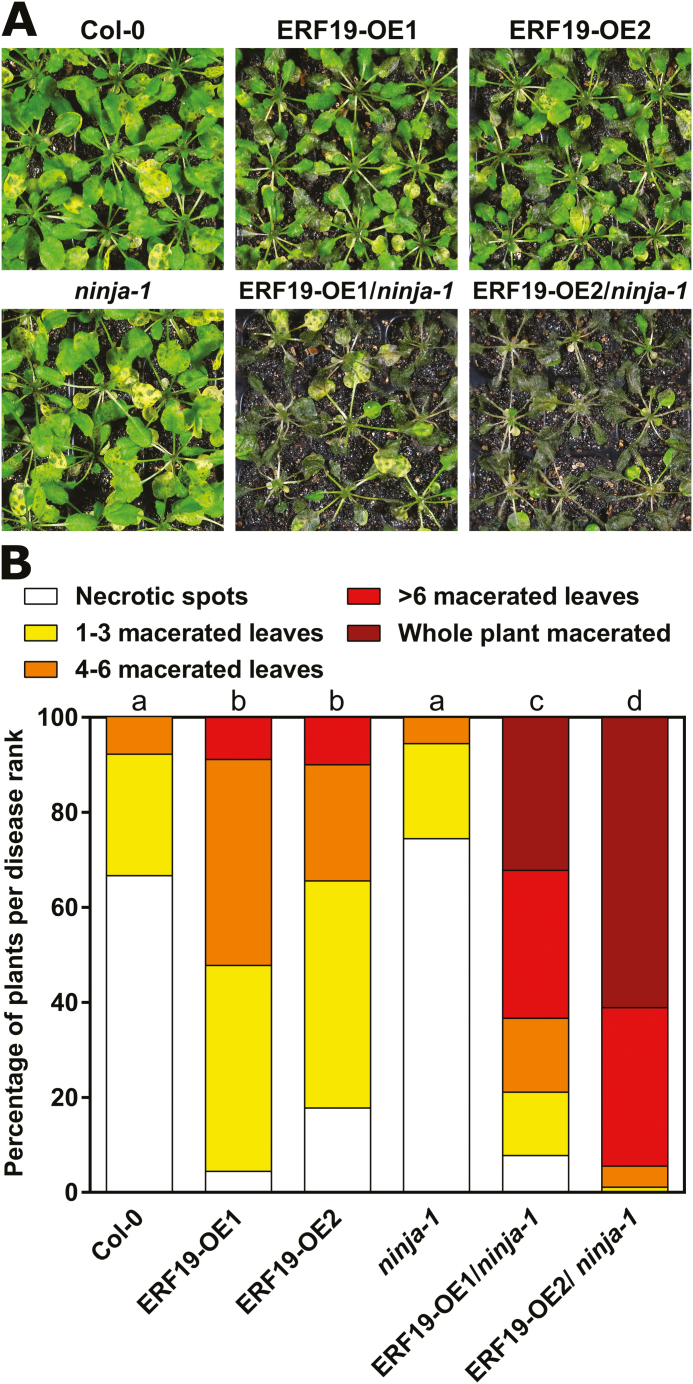
Hypersusceptibility to *B. cinerea* by *ERF19* overexpression is enhanced in *ninja-1*. (A and B) *B. cinerea* resistance in transgenic lines overexpressing *ERF19*. Four-week-old plants were spray inoculated with a *B. cinerea* spore suspension (10^5^ spores ml^–1^ in 1/4 PDB). Symptoms were photographed (A) and disease ranks were determined (B) at 5 dpi. Data in (B) represent 90 biological replicates (*n*=90) pooled from three independent experiments. The distribution of the disease rank proportions among the lines was analyzed using the χ^2^ test. Groups that do not share a letter are significantly different in the distribution of disease ranks (*P*<0.01).

Since only NINJA strongly repressed ERF19 transactivation, we focused on studying the biological impact of NINJA on ERF19. To this end, disease resistance of the *NINJA* loss-of-function mutant *ninja-1* overexpressing *ERF19* was tested (Acosta *et al.*, 2013). Two ERF19-OEs/*ninja-1* independent lines overexpressing *ERF19-GFP* and with increased ERF19–GFP proteins, that demonstrated comparable expression levels to ERF19-OEs in the Col-0 background (Supplementary Fig. S8A, B), were selected for phenotypical analyses. The *ninja-1* mutant appeared to have a long petiole phenotype when grown in our laboratory conditions, and ERF19-OEs/*ninja-1* plants showed reduced rosette and leaf sizes (Supplementary Fig. S8C). We first evaluated the *B. cinerea* resistance of ERF19-OEs/*ninja-1* lines by droplet inoculation of *B. cinerea*. The *ninja-1* mutant developed slightly, but significantly smaller lesions than Col-0 WT plants (Supplementary Fig. S8D), and this may be due to the de-repression of the JA signaling pathway that contributes to *B. cinerea* resistance, in *ninja-1* (Gasperini *et al.*, 2015; Zhang *et al.*, 2017). In contrast, overexpression of *ERF19* in *ninja-1* reversed the resistant phenotype of *ninja-1* to susceptible levels comparable with ERF19-OEs (Supplementary Fig. S8D). We speculated that the droplet inoculation method did not faithfully reflect the susceptibility of ERF19-OEs/*ninja-1* to *B. cinerea* as the disease evaluation was limited by the leaf size, with ERF19-OEs/*ninja-1* being much smaller than the other lines (Supplementary Fig. S8C). The disease resistance of ERF19-OEs/*ninja-1* against *B. cinerea* was thus assessed through spray inoculation, and progression of *B. cinerea* was ranked according to disease symptoms. After spray inoculation with *B. cinerea* spores, ERF19-OEs/*ninja-1* lines developed dramatic disease symptoms. Most of the plants were indeed heavily or completely macerated at 5 dpi (Fig. 7A, B). In contrast, ERF19-OE plants exhibited only several macerated leaves, and symptoms were less severe than in ERF19-OEs/*ninja-1* (Fig. 7A, B). While the majority of the Col-0 and *ninja-1* plants developed symptoms with necrotic spots, they showed the least severe symptoms of the lines tested (Fig. 7A, B). These results indicate that a loss of *NINJA* function strongly enhanced susceptibility to *B. cinerea* in *ERF19* overexpression lines. Since overexpression of *ERF19* increased Arabidopsis sensitivity to *B. cinerea*, a further increase in sensitivity by a loss of *NINJA* function in ERF19-OEs/*ninja-1* plants implies that NINJA represses the function of ERF19 in Arabidopsis immunity against *B. cinerea*. In summary, our data based on biochemical and genetic approaches strongly suggest that NINJA associates and negatively regulates the function of ERF19 in Arabidopsis immunity.

## Discussion

### ERF19 negatively regulates PTI

**Fig. 8.**
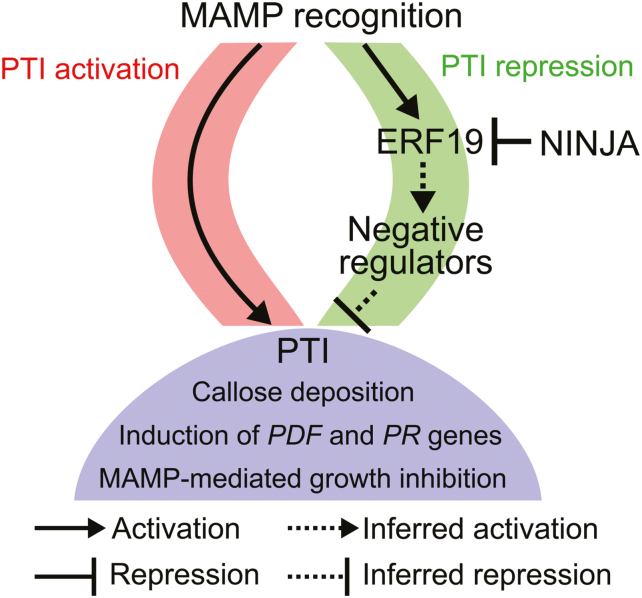
Proposed model for ERF19 and NINJA roles in PTI. MAMP perception initiates PTI repression signaling, triggering the induction of *ERF19*, in parallel with PTI activation signals. Accumulation of ERF19 may transcriptionally induce negative regulators of PTI, which are likely to be involved in the suppression of PTI signaling. PTI responses such as callose deposition, induction of *PDF* and *PR* genes, and MAMP-induced growth arrest are turned down by the ERF19-mediated pathway. The repressor NINJA provides another layer of control on PTI signaling through negative regulation of ERF19 function.

*ERF19* was first identified as one of the genes highly induced by chitin (Libault *et al.*, 2007) and is used as a marker for chitin elicitation (Fakih *et al.*, 2016). ERF19 is also involved in the regulation of plant growth, flowering time, and senescence, and positively regulates drought tolerance (Scarpeci *et al.*, 2017). Here we report that ERF19 functions as a negative regulator of Arabidopsis immunity. The fact that ERF19 positively regulates drought tolerance and negatively regulates immunity suggests a potential role for ERF19 in modulating the crosstalk between abiotic and biotic stress signaling pathways (Atkinson and Urwin, 2012). Notably, our phenotypic studies of ERF19-OEs and ERF19–SRDXs show that ERF19 negatively regulates disease resistance against the fungus *B. cinerea* and *Pst* DC3000 bacteria. Although ERF19-OEs exhibited curly leaves and reduced rosette size, the increased disease susceptibility of ERF19-OEs is probably not linked to the altered developmental habitus of *ERF19* overexpression. Indeed, we showed that ERF19-iOEs with appearance and morphology indistinguishable from those of the WT Col-0 were also hypersusceptible to *B. cinerea* when *ERF19* overexpression was induced by β-Est. These observations suggest that an altered plant growth pattern is not the major determinant of ERF19-mediated susceptibility. In line with this argument, small size plants, as a result of overexpression of TFs, could display either increased or decreased resistance against pathogens (Chen and Chen, 2002; Xing *et al.*, 2008; Tsutsui *et al.*, 2009), further suggesting that plant growth habitus is not a decisive measure of plant resistance. Importantly, the altered *B. cinerea* and *Pst* DC3000 resistance in ERF19-OEs and ERF19–SRDXs was correlated with an altered activation of PTI. PTI functions through common signaling pathways to activate transcriptionally defense responses against invading pathogens (Kim *et al.*, 2014). The necrotrophic fungus *B. cinerea* and the hemi-biotrophic bacterium *Pst* DC3000 are distinct microorganisms and therefore the observed altered resistance to different types of pathogens may be the result of perturbations of a broad spectrum immunity such as the PTI signaling network. Up-regulation of MAMP-specific marker genes was indeed repressed in ERF19-OEs and enhanced in ERF19–SRDXs, suggesting that ERF19 negatively regulates the PTI signaling network. In addition, *ERF19* was induced by fungal and bacterial MAMPs, and the diverse natures of these MAMPs further imply that ERF19 is a critical, downstream regulator in a common, general PTI signaling network. Since ERF19 acted as a transcriptional activator when analyzed by PTA assays and PTI was negatively correlated with ERF19 function (ERF19-OE versus ERF19–SRDX), we propose that the repression of PTI signaling by ERF19 is likely to be mediated through the transcriptional activation of negative regulators of PTI. These negative regulators, which may consist of repressors, co-repressors, kinases, phosphatases, E3 ligases, histone modification enzymes, and miRNAs (Couto and Zipfel, 2016; Li *et al.*, 2010; Schwessinger and Zipfel, 2008), could in turn transcriptionally, post-transcriptionally, and/or post-translationally suppress PTI signaling pathways (Fig. 8).

### Transcriptional regulation of *ERF19*

Rapid and transient up-regulation of *ERF19* by pathogens and MAMPs may seem paradoxical, since ERF19 plays a negative role in PTI activation. In fact, positive and negative regulators of immunity work in concert to mount appropriate levels of defense responses (Couto and Zipfel, 2016). In line with this, *ERF4*, *ERF9*, rice *OsERF922*, and potato *StERF3* are induced by pathogens and function as negative regulators in plant immunity (McGrath *et al.*, 2005; Liu *et al.*, 2012; Maruyama *et al.*, 2013; Tian *et al.*, 2015). In addition, the *L-type lectin receptor kinase-V.5* (*LecRK-V.5*), which is induced specifically in stomatal guard cells by *Pst* DC3000 and flg22, negatively regulates pathogen- and MAMP-induced stomatal closure, a common response of PTI (Melotto *et al.*, 2006; Arnaud *et al.*, 2012; Desclos-Theveniau *et al.*, 2012). Furthermore, flg22-induced *WRKY18* and *WRKY40* act redundantly to regulate flg22-triggered genes negatively (Birkenbihl *et al.*, 2017*b*). Collectively, these studies show that recognition of pathogens or MAMPs can transcriptionally induce negative regulators of immunity, which are necessary to buffer plant defense outputs.

The SA, JA, and ET pathways are known to play important roles in regulating the pathogen-induced TF network. For example, expression of *ERF1* after *Fusarium oxysporum* f. sp. *conglutinans* inoculation depends on JA and ET signaling pathways and is independent of SA (Berrocal-Lobo and Molina, 2004). Similarly, *B. cinerea*-induced *ERF96* requires intact JA and ET pathways (Catinot *et al.*, 2015). In contrast, JA and ET signaling negatively regulate *Pst*-induced *WRKY48* (Xing *et al.*, 2008). By using appropriate mutants, we showed that rapid induction of *ERF19* by chitin was unaffected when SA, JA, and ET signaling were individually impaired. It is possible that SA, JA, and ET act redundantly in the transcriptional control of chitin- (or MAMP-) induced *ERF19* so that the loss of one defense pathway is compensated by other functional signaling pathways. Indeed, it has been shown that the transcriptional network of PTI signaling is highly buffered, robust, and tunable (Kim *et al.*, 2014; Hillmer *et al.*, 2017). The induction of *ERF19* by chitin (or MAMPs) could also be regulated in addition to or independently of SA, JA, and ET.

### ERF19 buffers MAMP-induced growth inhibition

Plant growth and immunity are maintained at a fine balance to ensure plant survival. In the presence of invading pathogens, positive and negative regulators of immunity together tailor this balance to ensure appropriate levels of defense outputs. Exaggerated defense responses that tip the balance towards immunity can hamper plant growth and survival. For example, constitutive activation of ERF6 or overexpression of *ERF11* results in direct activation of defense genes, but these transgenic plants suffer from severe growth defects (Tsutsui *et al.*, 2009; Meng *et al.*, 2013). In addition, the L-type lectin receptor kinase-VI.2 (LecRK-VI.2) associates with FLS2 and functions as a positive regulator of PTI (Singh *et al.*, 2012; Huang *et al.*, 2014). Plants with high expression of *LecRK-VI.2* show constitutive PTI responses but display a dwarf phenotype (Singh *et al.*, 2012). Furthermore, loss of *BAK1-INTERACTING RECEPTOR-LIKE KINASE 1* (*BIR1*), a negative regulator of plant immunity, leads to constitutive activation of defense responses and cell death, which dramatically hampers plant growth (Gao *et al.*, 2009). These studies illustrate that genetic disruption of crucial immune regulators can deleteriously affect plant growth. Although ERF19 functions as a negative regulator of PTI, unlike the *bir1* mutant (Gao *et al.*, 2009), the ERF19–SRDX lines showed WT growth under normal conditions and did not exhibit constitutive activation of PTI responses. The dominant repressor *ERF19–SRDX* was regulated by the native promoter of *ERF19*. This basal expression of *ERF19–SRDX* might thus be insufficient to trigger constitutive PTI activation. In spite of normal growth, flg22- or elf18-induced growth inhibition was much more severe on ERF19–SRDX lines than on Col-0 WT, even at low concentrations of flg22 or elf18. The high sensitivity of ERF19–SRDXs to MAMP-mediated growth arrest implies that in response to MAMPs, ERF19 acts as a buffering regulator to prevent exaggerated growth arrest, which could negatively impact plant growth. In agreement with this, ERF19-OEs showed diminished growth inhibition imposed by high concentration of MAMPs. Taken together, our data suggest that ERF19 is part of a buffering mechanism to avoid exaggerated PTI activation and MAMP-mediated growth arrest to maintain a proper balance between growth and immunity upon MAMP recognition.

### NINJA negatively regulates ERF19

Post-translational regulation such as protein–protein interaction is known to alter the transcriptional activities of TFs (Licausi *et al.*, 2013). For example, EIN3 and MYC2, a crucial TF regulating JA signaling, interact and reciprocally affect each other’s functions (Song *et al.*, 2014; Zhang *et al.*, 2014). In addition, JAZ1 and JAZ proteins negatively regulate the functions of EIN3 and MYC TFs, respectively (Chini *et al.*, 2007; Pauwels and Goossens, 2011; Zhu *et al.*, 2011; Zhang *et al.*, 2015). Such negative regulations are thought to modulate fine-tuning mechanisms to achieve rigorous transcriptional controls. NINJA was originally identified as the adaptor between JAZ proteins and the transcriptional co-repressors TPL and TPRs and was demonstrated to act as a negative regulator of JA signaling (Pauwels *et al.*, 2010). Later studies showed that NINJA is also involved in the regulation of root growth (Acosta *et al.*, 2013; Gasperini *et al.*, 2015) and, together with topoisomerase II-associated protein PAT1H1, NINJA participates in the maintenance of root stem cell niche (Yu *et al.*, 2016). In this study, we found a novel function for NINJA in the negative regulation of ERF19. The repression mechanism(s) of NINJA on ERF19 may be linked to ERF19 association with NINJA that in turn recruits other co-repressors such as TPL (Pauwels *et al.*, 2010), and thus suppresses the transcription of the ERF19-bound loci. In addition, association with NINJA may change the conformation of ERF19 and subsequently inhibit the transcriptional function of ERF19 as observed in MYC3–JAZ9 regulation (Zhang *et al.*, 2015). Such a conformational change may hinder the ability of ERF19 to recruit co-activators and/or to bind to DNA. Our data provide evidence that NINJA is involved in the regulation of ERF19 function and further suggest that through modulation of ERF19 at transcriptional and post-translational levels, plants can fine-tune PTI to cope with the vast variety of environmental stimuli they face.

## Supplementary Data

Supplementary data are available at *JXB* online.

Fig. S1. Characterization of the HA-ERF19 line.

Fig. S2. Characterization of lines overexpressing *ERF19*.

Fig. S3. *B. cinerea*-mediated lesions in ERF19-iOE lines.

Fig. S4. Growth phenotypes of ERF19-OE and ERF19-iOE lines.

Fig . S5. Time course study of *ERF19* expression after treatment with 200 µg ml^–1^ chitin, water, or 1/2 MS.

Fig. S6. Expression of PTI marker genes in ERF19-OEs.

Fig. S7. Characterization of ERF19–SRDXs.

Fig. S8. Characterization of ERF19-OEs/*ninja-1*.

Table S1. Primers used in this study.

## Author contributions

P-YH and LZ designed the research; P-YH performed most experiments; Y-PL started the project by screening the *At*TORF-Ex collection; JZ generated ERF19-iOE lines and performed Co-IP analyses; CC performed the time course study of ERF19 expression after mock treatments; BJ conducted Y2H and BiFC experiments and rough phenotyping of ERF19–SRDX and ERF19-OE/*ninja-1* lines; J-HY performed flg22-induced *ERF19* analysis; KC cloned *ERF19-SRDX* and generated ERF19–SRDX lines. P-YH and LZ analyzed the data and wrote the manuscript; and LZ supervised the project.

## Supplementary Material

Supplementary Figures S1-S8 and Table S1
