# Supplementary material for: NINJA-associated ERF19 negatively regulates Arabidopsis pattern-triggered immunity
Source: J Exp Bot. 2018 Nov 21;70(3):1033–47. doi: 10.1093/jxb/ery414 (PMC6363091; doi:10.1093/jxb/ery414)
Supplement: Supplementary Figures S1-S8 and Table S1 [file ery414_suppl_supplementary-figures-s1-s8_table-s1.pdf]

NINJA-Associated ERF19 Negatively Regulates Arabidopsis Pattern-Triggered Immunity. *Pin-Yao Huang, Jingsong Zhang, Beier Jiang, Ching Chan, Jhong-He Yu, Yu-Pin Lu, KwiMi Chung , and Laurent Zimmerli*

Supplementary Figures S1–S7 and Table S1

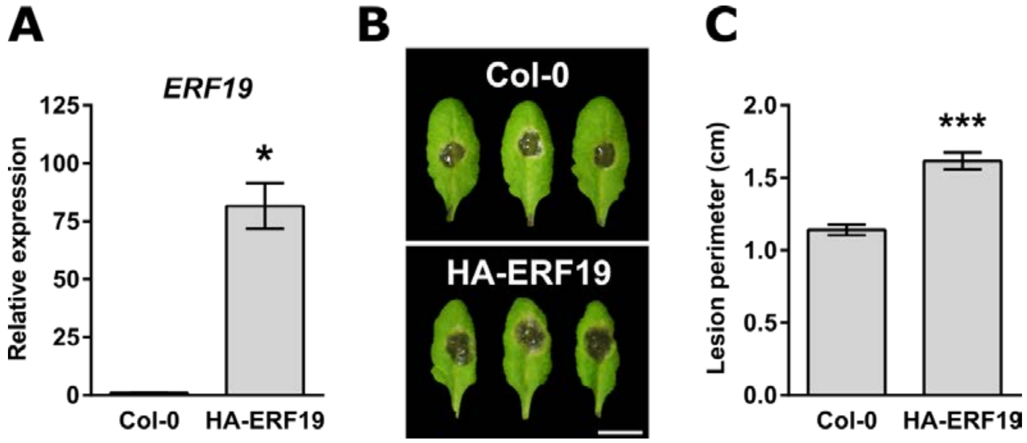

Fig. S1 Characterization of the HA-ERF19 line.

(A) *ERF19* expression in 12-day-old HA-ERF19 seedlings was determined by qRT-PCR. *UBQ10* was used for normalization. Relative gene expression levels were compared to Col-0 (defined value of 1). Data represent mean ± SD of three replicates (n = 3). Asterisk indicates a significant difference to Col-0 based on a *t* test (*$P < 0.05$).

(B) Disease phenotypes of HA-ERF19 against *B. cinerea*. Leaves of 5-week-old plants were droplet-inoculated with 8 µL of *B. cinerea* spore suspension (10^5 spores mL^{-1} in 1/4 PDB). Disease symptoms were photographed at 3 days post inoculation (dpi).

(C) *B. cinerea*-mediated lesion perimeters in HA-ERF19. Data represent average ± SE from at least 48 lesion perimeters (n > 48), pooled from 3 independent experiments. Asterisks indicate a significant difference to Col-0 based on a *t* test (***$P < 0.001$).

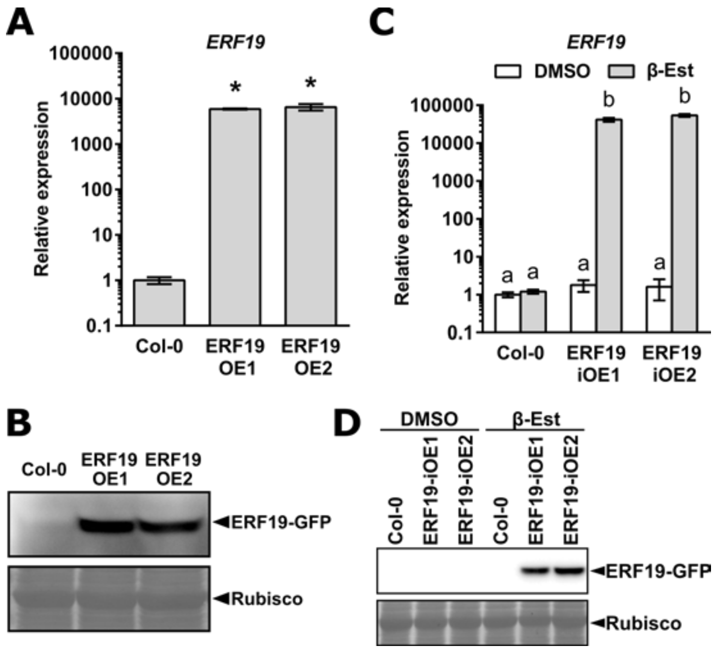

Fig. S2 Characterization of lines overexpressing *ERF19*.

(A,C) *ERF19* expression in 12-day-old seedlings was determined by qRT-PCR in ERF19-OEs (A) and ERF19-iOEs (C). *UBQ10* was used for normalization. Relative gene expression levels were compared to Col-0 (A) or DMSO-treated Col-0 (C) (defined value of 1). Data represent mean ± SD of three replicates (n = 3). Asterisks indicate a significant difference to Col-0 based on a *t* test (**P* < 0.05)(A), and different letters denote significant differences between groups based on a two-way ANOVA analysis (*P* < 0.01)(C).

(B,D) Expression of ERF19-GFP in 12-day-old ERF19-OEs (B) and ERF19-iOEs (D) was determined by immunoblot with an anti-GFP antibody (top panel). Coomassie blue staining of Rubisco is shown to assess equal loading in each lane (bottom panel). For analyses of ERF19-iOEs, seedlings were treated with 20 μM β-Est or DMSO 24 h before downstream experiments. Experiments were performed 3 times independently with similar results.

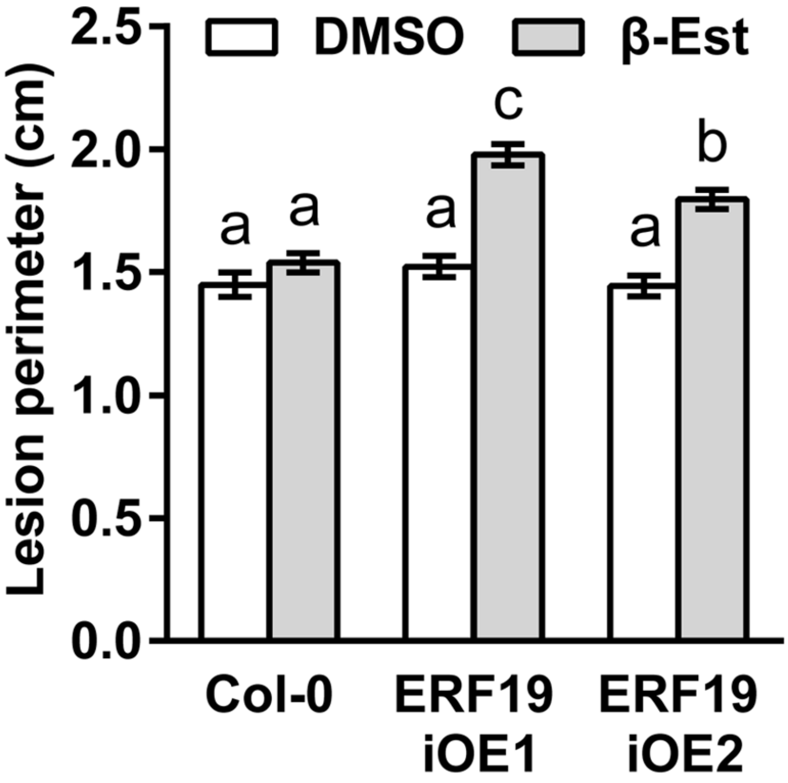

Fig. S3 *B. cinerea*-mediated lesions in ERF19-iOE lines.

Leaves of 5-week-old ERF19-iOEs were droplet-inoculated with 8 µL of *B. cinerea* spore suspension (10^5 spores mL^{-1} in 1/4 PDB) 24 h after treatment with 20 µM β-Est or DMSO as control. Lesion perimeters were measured at 3 dpi. Data represent average ± SE of at least 80 lesion perimeters pooled from 3 independent experiments each with at least 6 plants per line. Different letters denote significant differences between groups based on a two-way ANOVA analysis ($P < 0.01$).

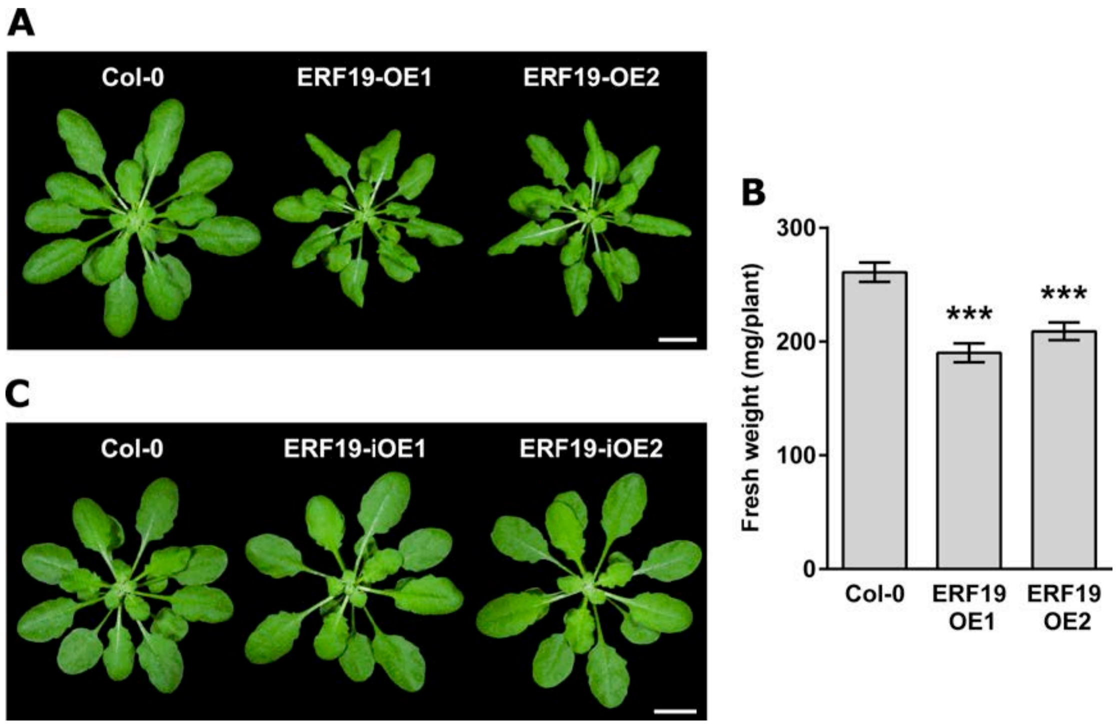

Fig. S4 Growth phenotypes of ERF19-OE and ERF19-iOE lines.

(A) Representative rosettes of 5-week-old Col-0 and ERF19-OEs. Scale bar represents 1 cm.

(B) Fresh weight of 5-week-old Col-0 ERF19-OEs. Data are average fresh weight per plant ± SE of 36 biological replicates (n = 36) from 4 independent experiments. Asterisks indicate a significant difference to Col-0 based on a *t* test (***$P < 0.001$).

(C) Representative rosettes of 5-week-old Col-0 and ERF19-iOEs. Scale bar represents 1 cm.

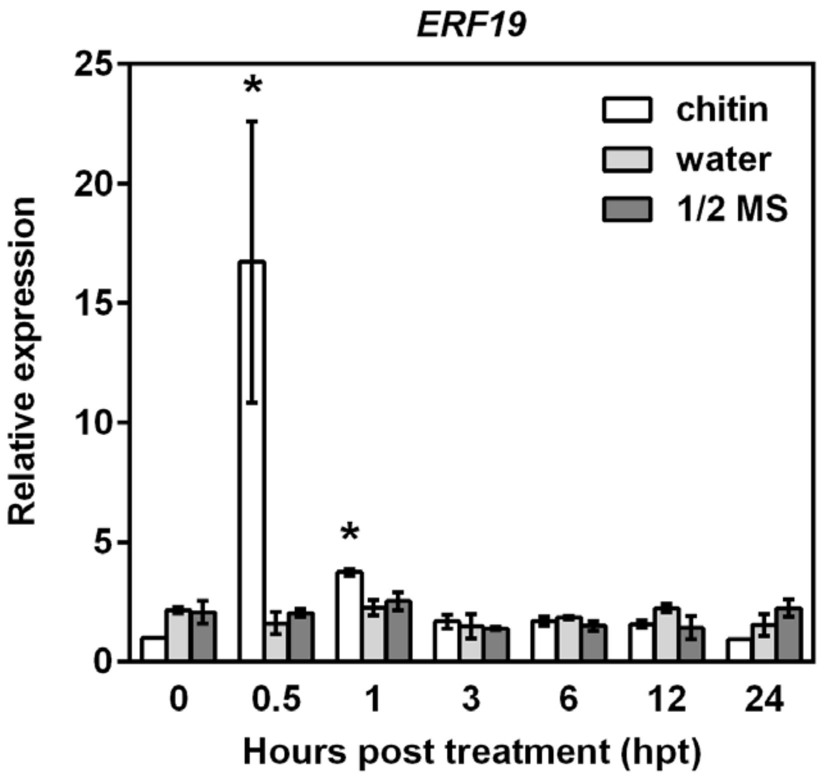

Fig. S5 Time course study of *ERF19* expression after treatment with 200 µg mL^{-1} chitin, water, or ½ MS.

Twelve-day-old seedlings were collected at indicated time points, and *ERF19* expression was determined by qRT-PCR. After normalization with *UBQ10*, *ERF19* expression levels were compared to chitin treatment at time 0 (defined value of 1). Data represent mean ± SD of eight replicates (n = 8) from two independent experiments. Asterisks denote values significantly different from the chitin-treated sample at time 0 based on a two-way ANOVA (*P < 0.05).

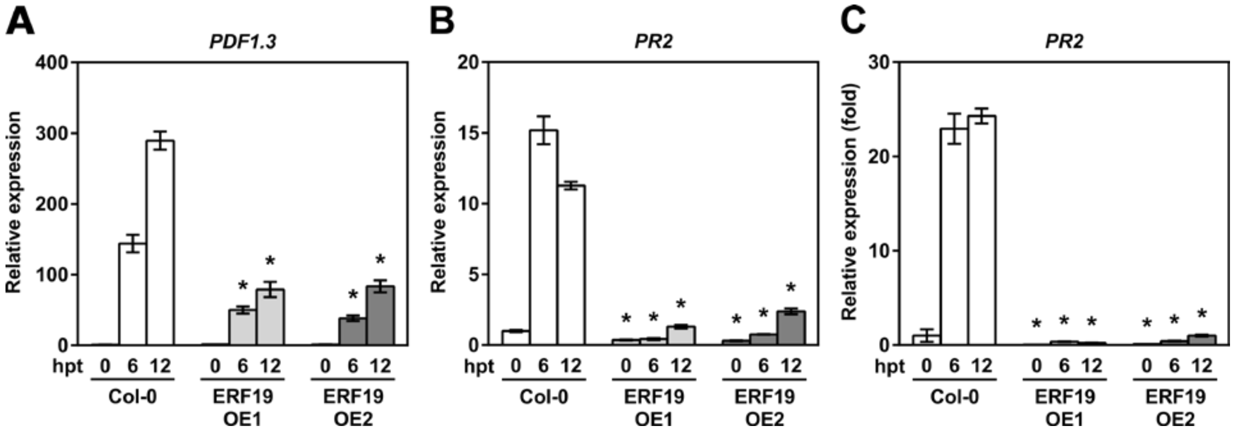

Fig. S6 Expression of PTI marker genes in ERF19-OEs.

(A-C) Chitin-induced *PDF1.3* (A), flg22-induced *PR2* (B), and elf18-induced *PR2* (C) in ERF19-OEs were determined by qRT-PCR. Twelve-day-old seedlings were treated with 200 µg mL^{-1} chitin, 1 µM flg22, or 1 µM elf18. Samples were collected at indicated time points, and *UBQ10* was used for normalization. Relative gene expression levels were compared to Col-0 at time 0 (defined value of 1). Data represent mean ± SD of three replicates (n = 3). Asterisks denote values significantly different from respective Col-0 controls based on a *t* test (*P < 0.05).

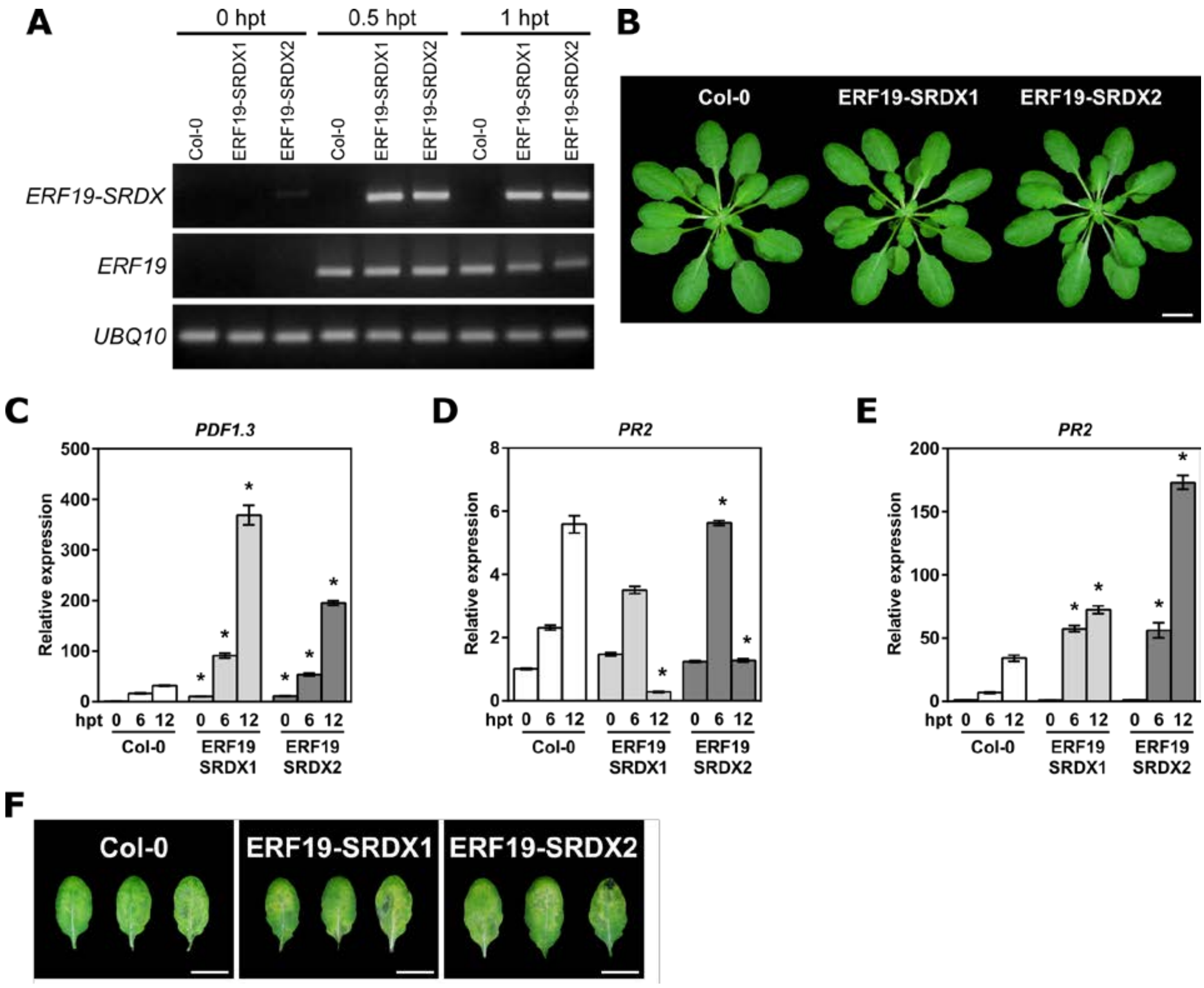

Fig. S7 Characterization of ERF19-SRDXs.

(A) Expression of *ERF19-SRDX* in transgenic ERF19-SRDXs. Expression of *ERF19-SRDX* is controlled by the native promoter of *ERF19*. Expression levels of the transgene *ERF19-SRDX* and the endogenous *ERF19* in 12-day-old seedlings were analyzed by RT-PCR. Seedlings were treated with 200 µg mL^{-1} chitin for 0.5 or 1 hour to induce *ERF19-SRDX* and *ERF19* expression. *UBQ10* was used as a loading control. This experiment was performed 3 times with similar results.

(B) Representative rosettes of 5-week-old Col-0 and ERF19-SRDXs. Scale bar represents 1 cm.

(C-E) Activation of PTI marker genes in ERF19-SRDXs. Chitin-induced *PDF1.3* (C), flg22-induced *PR2* (D), and elf18-induced *PR2* (E) in ERF19-SRDXs were determined by qRT-PCR. Twelve-day-old seedlings were treated with 200 µg mL^{-1} chitin, 1 µM flg22, or 1 µM elf18. Samples were

collected at indicated time points, and *UBQ10* was used for normalization. Relative gene expression levels were compared to Col-0 WT at time 0 (defined value of 1). Data represent mean ± SD of three replicates (n = 3). Asterisks denote values significantly different from respective Col-0 controls based on a *t* test (*P < 0.05).

(F) *Pst*-mediated disease symptoms in ERF19-SRDXs. Five-week-old plants were dip-inoculated with 10^6 cfu mL^{-1} *Pst*. Photographs were taken at 3 dpi. Scale bars represent 1 cm.

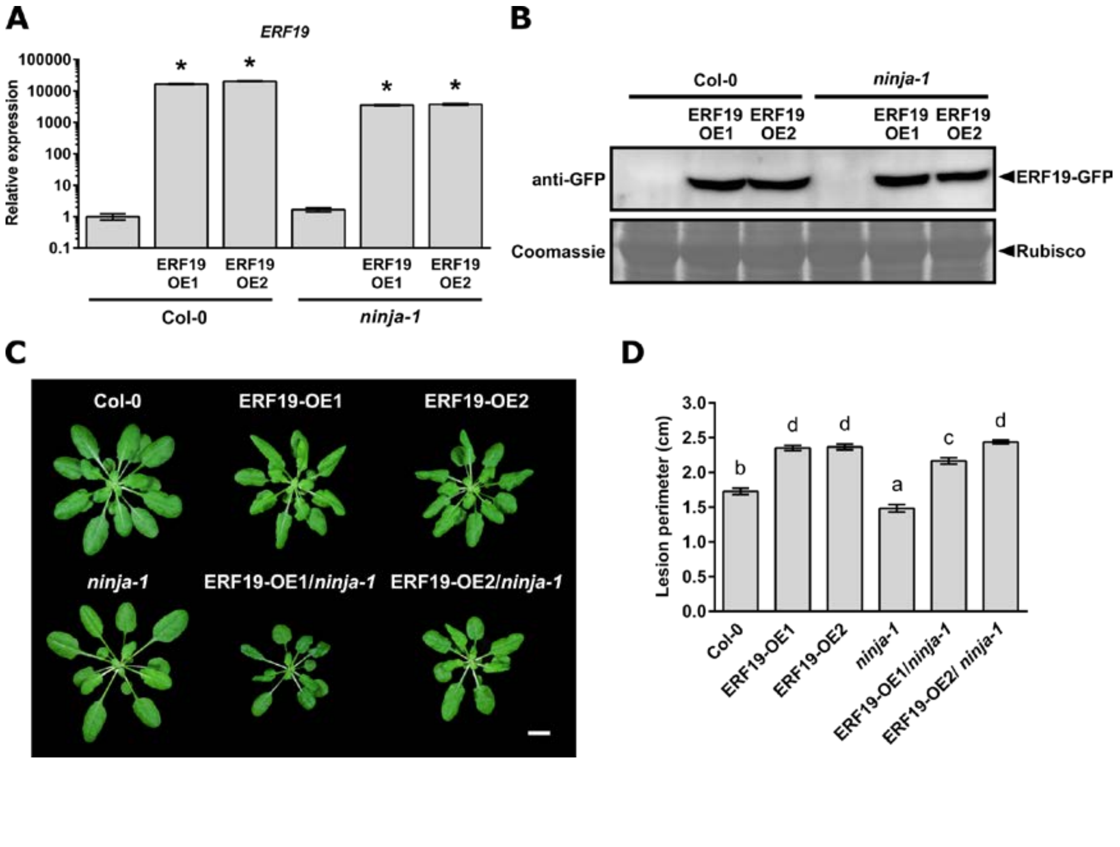

Fig. S8 Characterization of ERF19-OEs/*ninja-1*.

(A) *ERF19* expression in the transgenic lines overexpressing *ERF19*. Levels of *ERF19* transcripts in 12-day-old seedlings were determined by qRT-PCR. *UBQ10* was used for normalization. Relative gene expression levels were compared to Col-0 (defined value of 1). Data represent mean ± SD of three replicates (n = 3). Asterisk indicates a significant difference to Col-0 based on a *t* test (**P* < 0.05).

(B) ERF19-GFP expression in the transgenic lines overexpressing *ERF19*. Total proteins extracted from 12-day-old seedlings were analyzed by immunoblotting with an anti-GFP antibody (top panel). Coomassie blue staining of Rubisco is shown to assess equal loading in each lane (bottom panel). Experiments were performed 3 times with similar results.

(C) Representative rosettes of 5-week-old Col-0, ERF19-OEs, *ninja-1*, and ERF19-OEs/*ninja-1*. Scale bar represents 1 cm.

(D) *B. cinerea*-mediated lesions in ERF19-OEs/*ninja-1* lines. Leaves of 5-week-old plants were droplet-inoculated with 8 µL of *B. cinerea* spore suspension (10^5 spores mL^{-1} in 1/4 PDB). Lesion perimeters were measured at 3 dpi. Data represent average ± SE of at least 54 lesion perimeters pooled from 3 independent experiments each with at least 6 plants per line. Different letters denote significant differences between groups based on a one-way ANOVA analysis ($P < 0.05$).

Table S1

Primers used in this study. Nucleotides underlined indicate restriction sites.

Purpose	Primer name	Sequence (5' - 3')
To clone pCR8-ERF19	ERF19-F1	ATGGATTACAGAGAATCCACCGGTGA
	ERF19-R1	AACGTGATCGTGGCCGCCCA
To clone pBCKH-ERF19-SRDX	ERF19-F2	CCCGGCGCGCCATGTAGAAAGAGACTCCTTT
	ERF19-R2	GGGCCCGGGAACGTGATCGTGGCCGCCCA
To clone GAL4DB-ERF19	ERF19-F3	CTAGCCCGGGGATGGATTACAGAGAATCC
	ERF19-R3	ATGCGTCGACTCAAACGTGATCGTGGC
To clone GAL4DB-ERF19-SRDX	SRDX-R	TTAAGCGAAACCCAAACGGAGTTC
	pGAL4-F	CCAAGCTAATTCCGGGCG
	pGAL4-R	ATGAGTCAAAGCTGGGTATTG
To clone pCR8-NINJA	NINJA-F	ATGGCAGATGATAATGGGCTCGA
	NINJA-R1 (with stop codon)	TCAGGTGTGAGCTGACGCTGCA
	NINJA-R2	GGTGTGAGCTGACGCTGCA
To clone pCR8-HDA6	HDA6-F	ATGGAGGCAGACGAAAGC
	HDA6-R1 (with stop codon)	TTAAGACGATGGAGGATTCACGT
	HDA6-R2	AGACGATGGAGGATTCACGTC
To clone pCR8-HDA19	HDA19-F	ATGGATACTGGCGGCAATTC
	HDA19-R1 (with stop codon)	TTATGTTTTAGGAGGAAACGCCTG
	HDA19-R2	TGTTTTAGGAGGAAACGCCTG
To clone 35S:ERF19-HA$_3$:NOS	35S-F	CACCTCAAGTCGGTGACGGTGATA
	NOS-R	CCCAAAAACAACCTCTCCAA
To clone the nuclear marker construct	NLS-mCherry-F	CACCATGCCGAAGAAGAAGCGCAAGGTGAGCAAGGGCGAGGAGGAT
	mCherry-R	CCCGGGCTTGTACAGCTCGTCCAT
	mCherry-F	AGCAAGGGCGAGGAGGAT

	mCherry-NLS-R	TCACACCTTGCGCTTCTTCTTCGGCTTGTACAGCTCGTCCATG
To analyze *ERF19-SRDX* (ERF19-F4 and SRDX-R4) or endogenous *ERF19* (ERF19-F4 and ERF19-R5) by RT-PCR	ERF19-F4	GGATGCGGAGATACGACGAC
	SRDX-R4	AAGCGAAACCCAAACGGAGT
	ERF19-R5	TTAGCACCACACCTGTTTTGA
For RT-qPCR analysis	UBQ10-qF	GGCCTTGTATAATCCCTGATGAAT
	UBQ10-qR	AAAGAGATAACAGGAACGGAAACA
	ERF19-qF	GAAATGGGGCAAATGGGTATC
	ERF19-qR	CTGCTGTTGAGAATGAACCTAA
	PDF1.2-qF	AATCTTTGGTGCTAAATCGTGTG
	PDF1.2-qR	CAACGGGAAAATAAACATTAAAA
	PDF1.3-qF	AGAAGTTGTGCGAGAAGCCA
	PDF1.3-qR	ACACACCATGAAGCACCAAGT
	PR1-qF	GGGAAAACTTAGCCTGGGGT
	PR1-qR	CACTTTGGCACATCCGAGTC
	PR2-qF	TGCAGAACATCGAGAACG
	PR2-qR	TACTCATCCCTGAACCTTCC
